# Evolution of Hypofractionated Accelerated Radiotherapy for Prostate Cancer – The Sunnybrook Experience

**DOI:** 10.3389/fonc.2014.00313

**Published:** 2014-11-14

**Authors:** Hima Bindu Musunuru, Patrick Cheung, Andrew Loblaw

**Affiliations:** ^1^Odette Cancer Centre, Sunnybrook Health Sciences Centre, Toronto, ON, Canada; ^2^Department of Radiation Oncology, University of Toronto, Toronto, ON, Canada; ^3^Department of Health Policy, Measurement and Evaluation, University of Toronto, Toronto, ON, Canada

**Keywords:** prostate cancer, image-guided radiotherapy, stereotactic ablative radiotherapy, quality of life

## Abstract

Stereotactic ablative body radiotherapy (SABR) is a newer method of ultra hypo fractionated radiotherapy that uses combination of image-guided radiotherapy (IGRT) and intensity-modulated radiotherapy (IMRT) or volumetric modulated arc therapy (VMAT), to deliver high doses of radiation in a few fractions to a target, at the same time sparing the surrounding organs at risk (OAR). SABR is ideal for treating small volumes of disease and has been introduced in a number of disease sites including brain, lung, liver, spine, and prostate. Given the radiobiological advantages of treating prostate cancer with high doses per fraction, SABR is becoming a standard of care for low and intermediate-risk prostate cancer patients based upon the results from Sunnybrook and also the US-based prostate SABR consortium. This review examines the development of moderate and ultra hypo-fractionation schedules at the Odette Cancer centre, Sunnybrook Health Sciences. Moderate hypo-fractionation protocol was first developed in 2001 for intermediate-risk prostate cancer and from there on different treatment schedules including SABR evolved for all risk groups.

## Introduction

Prostate cancer is the most prevalent male cancer in the western world with varying management options as per the individual’s risk stratification, functional domain, and preference.

There is now convincing evidence that radiotherapy dose escalation is beneficial across all risk groups of prostate cancer patients (1, 2). Dose escalation beyond 81 Gy with conventional radiotherapy techniques has posed problems due to the increase in bowel toxicity (2) despite the use of image guided (IGRT) and intensity-modulated radiotherapy (IMRT).

Alternative means of safe radiotherapy dose escalation are low dose rate (LDR) or high-dose rate (HDR) brachytherapy alone or in combination with EBRT. While LDR brachytherapy monotherapy is established for low risk and selective intermediate-risk patients, HDR monotherapy remains experimental (3). HDR boost in combination with EBRT is an excellent example of safe radiotherapy dose escalation (4). Despite this being an appealing option, limitations include patient fitness for anesthetic exposure, urinary morbidity, and limited operating room capacity.

One notion that has revolutionized the field of radiotherapy has been the purported low α/β ratio for prostate cancer (5). Miralbell et al. presented the outcomes of 5969 patients, which calculated the α/β to be 1.3, 1.6, and 1.8 for low, intermediate, and high-risk prostate cancers (6). This in association with a higher α/β ratio for acute (10) and late (≈3–4) responding tissues, can theoretically improve the therapeutic ratio of hypofractionated radiotherapy (7). Strategies include either moderate hypo-fractionation (2–3.5 Gy per fraction) or ultra hypo-fractionation (>3.5 Gy per fraction).

These concepts have led to the development of various prostate hypofractionated accelerated radio therapy (pHART) protocols at the Odette Cancer Centre, Sunnybrook Health Sciences.

This is the first report describing the evolution of various gantry-based coplanar radiotherapy techniques for hypofractionation, adapted in accordance with the emerging radiotherapy technology. This review also discusses the role of hypo-fractionation in various prostate cancer risk groups and is the first to include a significant number of high-risk patients.

## Moderate Hypofractionation

### pHART1 – intermediate risk

In 2001, pHART1 was developed to treat 33 intermediate-risk prostate cancer patients. The first phase involved delivering 42 Gy in 21 fractions using three-dimensional conformal radiotherapy (3DCRT) to the prostate and proximal seminal vesicles with a 10-mm planning target volume (PTV) margin. Following CT simulation, patients had digital fluoroscopic imaging of the gold seed fiducials to quantify the respiratory induced prostatic motion. For the first nine fractions, location of the implanted fiducials was captured pre- and post-treatment using electronic portal imaging device (EPID). Based on this data, a patient specific PTV margin was derived and applied to the second phase of treatment, which was an IMRT boost using a dose of 30 Gy in 10 daily fractions with daily online EPID imaging.

The calculated mean PTV margin was 3 mm (range 2–5 mm) in the lateral direction, 3 mm (range 2–7 mm) in the superior–inferior, and 4 mm (range 2–8 mm) in the anterior–posterior directions. Three patients (9.1%) developed acute grade 3 urgency/frequency. This study showed that the intra-fraction prostate movement was generally small, allowing for significant PTV margin reduction if daily online imaging is performed (8).

### pHART2 – high risk

In 2004, 67 high-risk prostate cancer patients were enrolled into a prospective, phase 1 study (pHART2) delivering 67.5 Gy to the prostate in 25 fractions (2.7 Gy per day), while the pelvic nodes received 45 Gy in 25 fractions over the same period, in conjunction with 3 years of ADT. Patients were treated using a simple four field box technique (4FB) to deliver the pelvic elective nodal irradiation (ENI), while an optimized IMRT plan was used to deliver the concomitant IMRT boost to the prostate. The acute toxicity from this study has been very favorable (9).

In the next phase, the same dose-fractionation scheme was employed for the same high-risk group in another 30 additional patients. However, the radiotherapy technique was changed to deliver both ENI and concomitant prostate boost using a single optimized IMRT plan. The toxicity data has been updated for these 97 patients in this phase I–II study with a median follow up of 39 months. The incidence of acute grade 2 gastrointestinal (GI) toxicity was 37% (no grade 3+); acute genitourinary (GU) toxicity was 39% grade 2 and 4% grade 3. The late GI toxicity was 7% grade 2 (no grade 3+) and late GU toxicity was 5% grade 2, 3% grade 3, and 1% grade 4. All severe toxicities (grade 3 or greater) had resolved at the last follow-up visit. The 4-year biochemical relapse-free survival (bRFS) rate was 90.5%. A quality of life (QOL) study in this cohort demonstrated a modest decline in urinary and bowel domain but this only led to mild distress in QOL (10, 11).

Between 2007 and 2010, a third phase of the study was completed to treat an additional 144 patients (total 241 patients) with the same dose-fractionation scheme. The acute toxicity results from this entire cohort demonstrated a reduction in acute grade 2 GI toxicity, from 30.9% in patients receiving ENI with a 4FB technique, to 7.2% in patients treated with IMRT and full bladder filling protocol (12).

A phase 2 RCT study was opened in 2011 testing hypofractionated schedule against conventional fractionation. Both schedules provided ENI (46 Gy in 23 fractions in the conventional arm, and 48 Gy in 25 fractions in the hypofractionated boost arm) along with 2–3 years of ADT. The conventional arm boosted the prostate using cone-beam IGRT to a total prostate dose of 78 Gy (in 39 fractions) while the hypofractionated arm used gold seed fiducial IGRT and simultaneously boosted the prostate to a dose of 68 Gy in 25 fractions. The study is currently open and has accrued 64 patients to date (accrual goal 178).

In a recent systematic review of different hypo-fractionation schedules, the bRFS was 73% (range, 3.3–95.4%) at a median follow up of 36.5 months in the hypofractionated radiotherapy cohort with less acute and late toxicities, compared with a median bRFS of 66% (range, 34–79%) for the standard radiotherapy group (13). In the Italian hypofractionated RCT, 168 patients with intermediate and high-risk disease received a total of 9 months of ADT and were randomized to 62 Gy in 20 fractions in 4 weeks or 80 Gy in 40 fractions over 8 weeks (14). At a median of 70 months follow-up, bRFS was not statistically significant between the two treatment cohorts (85 vs. 79%, *p* = 0.065). In the subgroup analysis, patients with PSA ≥20 ng/ml or Gleason 4 + 3 had better bRFS with the hypo-fractionation schedule (15).

### pHART4: Post-operative RT

A hypo-fractionation schedule was developed for patients requiring post-operative radiotherapy in 2009. Thirty patients with pT3 NX-0 M0, positive surgical margins and/or a rising PSA post-radical prostatectomy (two consecutive rises in PSA, at least 4 weeks apart) were included in the pHART4 study. Adjuvant ADT was allowed at the discretion of the treating oncologist for up to 2 years.

Three gold seeds were inserted transperineally into the prostate bed using local anesthesia (one at urogenital diaphragm, one at each side of bladder neck). CTV was defined as per the widely accepted RTOG consensus guidelines (16). PTV was derived by adding a 4-mm symmetrical margin around the CTV (8). A dose of 51 Gy in 17 daily fractions was prescribed with the 95% isodose covering at least 99% of the PTV. Patients were treated using step-and-shoot IMRT (SS-IMRT) technique. This schedule was chosen to provide a biologically effective dose (BED) of 153 Gy, comparable to 154 Gy as delivered by 66 Gy in 2 Gy fractions (assuming an α/β of 1.5 Gy for prostate cancer).

At a median follow up of 24 months, only one patient had acute grade 3 GU toxicity (3%), with gross hematuria and clots. One patient developed greater than grade 1 (3%) late GU toxicity and 2 (6%) patients with >grade 1 late GI toxicity. Three patients (10%) had experienced biochemical failure, with two developing metastatic disease (17).

Recent data from two other groups suggest that hypo-fractionation is safe in the post-operative setting (18, 19).

## Ultra Hypofractionation

### pHART3: Low risk

A prospective, phase I/II ultra hypofractionated study (pHART3) using a schedule of 35 Gy in five (weekly) fractions was started in 2006 for low-risk prostate cancer patients. It was based on the hypothesis that the weekly treatment would allow maximal normal tissue repair without compromising tumor control, facts later confirmed by other groups (20).

The planning technique and 5-year results were described in detail in our previous reports (21, 22). Prostate was contoured as CTV and a 4-mm margin was added for PTV (8). Eighty four patients were treated using a SS-IMRT technique and daily image guidance using gold seed fiducials.

At a median follow up of 55 months (range 13–68 months), the 5-year bRFS was 98% (95% confidence interval of 96–100%) as per the Phoenix criteria (and 97% using ASTRO failure definition) (22). Acute GI toxicity was 0, 10, and 67% for grades 3, 2, and 1, while 1, 19, and 71% developed new grade 3, 2, and 1 GU toxicities (CTC v3.0 criteria), respectively. Four (5%) patients developed grade 2 GU late toxicity (RTOG criteria). Two required transurethral resection of the prostate and two required alpha antagonists. Three patients (7%) had hematochezia requiring treatment. One patient with a history of diverticulitis developed a rectocutaneous fistula post-radiation and refused definitive surgical correction (grade 4 toxicity).

These results are comparable to the outcomes from the stereotactic ablative body radiotherapy (SABR) multi-institutional consortium analysis. A median SABR dose of 36.25 Gy was delivered using robotic non-coplanar technique in 4–5 fractions either daily or on alternate days. At a median follow up of 36 months, the 5-year bRFS was 95% for low-risk patients. In the 104 (77% of 135 pts) low-risk patients with a minimum follow up of 5 years, bRFS was 99%. In the largest prostate cancer SABR series by Katz et al., acute (RTOG) and late (RTOG) ≥grade 2 GU/GI toxicities (acute Grade 2 GU-4%, GI-4%; Late Grade 2 GU-4%, and GI-2%) were more favorable in the 50 patients who had 35 Gy in five daily fractions when compared to phart 3 study. Late Grade 3+ GI toxicity was 1% in phart 3 and this was not observed in the Katz et al. study. It is difficult to conclude that there are clinically meaningful differences in toxicities between both techniques due to heterogeneity in patient factors and toxicity scoring criteria (23).

### pHART5: Intermediate risk

pHART5 protocol for intermediate-risk prostate cancer was developed in 2010, which hypothesized that HDR brachytherapy doses of 10–15 Gy can be delivered as a boost to the prostate with acceptable toxicity rates using SABR. The phase one component used MRI for prostate contouring, an intra rectal balloon, cone-beam CT (CBCT) image guidance, and volumetric modulated arc therapy (VMAT) for radiation delivery.

Phase 2 component began 2 weeks after the SABR boost. EPID images were taken before treatment is initiated using IMRT to deliver 2.5 Gy per day for 15 days delivering a total dose of 37.5 Gy. This unique dose-fractionation scheme is similar to the prospective benchmark HDR brachytherapy study conducted at Sunnybrook (24).

Ten patients in each cohort were each treated with an SABR boost of 10, 12.5, and 15 Gy. Patients tolerated the SABR boost very well with no acute grade 3 or higher toxicities seen. Patients had less acute GU toxicity from the SABR boost (even at the 15-Gy dose level) compared to the HDR boost (25). Ongoing follow-up will yield late toxicities.

Katz et al. reported favorable toxicity outcomes in 41 intermediate and 32 high-risk patients who received 45 Gy EBRT with a SABR boost (range 18–21 Gy in three fractions) (26). In a study by Collins et al., 24 patients were treated with SABR to a dose of 19.5 Gy in three fractions followed by IMRT to a dose of 50.4 Gy in 28 fractions. At a median follow up of 9.3 months, there was no acute toxicity ≥Grade 3 (27). There is emerging data that SABR plans can be optimized to reproduce the heterogeneity of HDR (28).

### pHART6: Low and intermediate risk

Oliai et al. demonstrated a dose response for intermediate and high-risk patients with the 3-year freedom from biochemical failure being 100% for the high-dose SABR group (37.5 Gy in five fractions) and 72% for the low dose group [35 or 36.25 Gy in five fractions, *p* = 0.03 (29)].This dose response was not maintained in the pooled analysis from the multi-institutional SABR consortium (30). In the study by Bolzicco et al., patients across all risk categories were treated with 35 Gy in five daily fractions using robotic non-coplanar technique. The 3-year bRFS was 94.4%, with four biochemical failures in intermediate and high-risk group patients (31).

Based on the toxicity profile of pHART3, a phase 2 prospective dose escalated SABR trial of 40 Gy in five fractions over 29 days for low and intermediate-risk prostate cancer patients was started in 2010. Using the linear quadratic equation with an α/β of 1.3 (6), a dose of 40 Gy in five fractions would be equivalent to 113 Gy delivered in 2 Gy fractions. For normal tissues such as rectum, assuming an α/β of 4, this would be equivalent to 80 Gy in 2 Gy fractions. This estimate may be conservative, given studies reporting the α/β of rectal tissue to be even higher (7). This dose is similar to our HDR + EBRT program (24) (EQD2 111 Gy, HDR 15 Gy with EBRT 37.5 Gy in 15 fractions), which demonstrated encouraging biochemical and toxicity outcomes. A similar benefit was also shown in the study by Taira et al., where EBRT at a dose of 45 Gy in 25 fractions was combined with LDR boost (I-125 (144 Gy) or Pd-103(108 Gy).The 12-year bRFS was 98.7, 95.9, and 90.4% for low, intermediate, and high-risk patients.

Treatment in phart 6 was delivered using SSIMRT technique using daily image guidance with gold seed fiducials and a 5-mm CTV-PTV margin. Thirty patients have been treated with a median follow-up of 24 months and no biochemical failures, grade 3+ acute or late toxicities have been observed (32). When phart 6 was compared with phart 3, there was no difference in grade + GU or GI toxicities but there were significant differences in QOL favoring the pHART3 protocol (smaller margin, lower dose), especially for the bowel bother domain (32).This is likely due to a dose and also volume effect, given the 5-mm margin. As a result of this, in our next generation SABR studies (phart 10 and phart 11), we have reduced the CTV-PTV margin to 3 mm and also reduced the PTV dose to match the phart 3 PTV dose (33.25 Gy). This reduction in margin was also facilitated by the change in treatment technique from SSIMRT (33) to VMAT (34).

### pHART7: Low and intermediate risk

Given that prostate cancer is a slow-growing malignancy, differences of 1–2 weeks in the overall treatment time between extreme hypofractionated radiotherapy schedules are unlikely to impact bRFS since treatment is completed within a short time (less than 30 days) (35, 36) but this has not been properly tested.

In contrast, small differences in treatment times can have impact on toxicity. In a study from Stanford delivering 36.25 Gy in five fractions, patients were initially treated in five consecutive days and the second cohort was treated on alternate days (20). Comparing the late GU and GI toxicities between two groups, the rate of grade 1–2 GU toxicity was 56 vs. 17%(*p* = 0.007) and grade 1–2 GI toxicity was 44 vs. 5%(*p* = 0.001) both favoring the every other day schedule. The difference in grade 3 GU toxicity was not statistically significant, likely due to small numbers (6 vs. 2%, *p* = 0.48) (37).

In this Canadian, multicentre, randomized phase II study (pHART7), 152 patients with low- and intermediate-risk prostate cancer were randomized to receive 40 Gy in five fractions over 29 days (one 8 Gy fraction per week) or over 11 days (one 8 Gy fraction every other day, excluding weekends). Entire prostate plus an additional 0.5 cm margin comprised PTV and treatment was delivered using the VMAT technique. The primary endpoint (acute QOL) results will be presented in early 2015.

### pHART10: Low and intermediate risk

As the next logical extension of SABR protocols, Odette cancer centre has developed a two-fraction SABR treatment protocol (pHART10) prescribing a dose of 26 Gy in two fractions to the prostate (CTV) and a PTV dose of 22 Gy in two fractions. Using an α/β ratio of 1.4 (6), this would be equal to EQD2 of 110 Gy to CTV (equivalent to our 40 Gy in five fraction SABR and our HDR/EBRT protocol) and 80 Gy to PTV (equivalent to 33.25 Gy in five fractions, the PTV dose in pHART3). The rectal and bladder EQD2 would be 55–62 Gy (using α/β ratios of 3–4).

The CTV–PTV margin is 3 mm. Due to the reduction in treatment time with VMAT over SSIMRT, the prostatic intra-fraction motion has reduced, which in turn facilitated the change in CTV-PTV margin from 5 mm (33) to 3 mm (34).

3D datasets and anterior rectal doses were acquired via optically stimulated luminescence (OSL) detectors held by a purpose-built intrarectal acrylic stem and stand (like an endorectal balloon). This protocol has been active since January 2014 and completed accrual within 8 months. Acute toxicity, prostate immobilization, and data about adequacy of PTV margins will be presented in early 2015.

### pHART8: High risk

The current standard of treatment for high risk locally advanced non-metastatic prostate cancer patients is combined T and ADT (38–40). Several EBRT dose escalation studies including standard and hypo-fractionation schedules have shown improvement in the 5-year bRFS in this cohort (2, 13, 15). LDR brachytherapy boost has shown excellent long-term results (41) and randomized data from the ASCENDE-RT study (dose escalated RT vs. LDR boost for high-intermediate or high-risk patients) is anticipated in 2015.

SABR was investigated by other investigators in the high-risk setting. Doses ranging from 35 to 37.5 Gy in five fractions using robotic non-coplanar technique (29, 31, 42, 43) and 43.84–45.2 Gy in eight fractions using gantry-based technique (44) have been described. The 6-year bRFS was 69% in the Katz et al. (42) cohort and the 3 year bRFS was 77.1% in the Oliai et al. group (29).

The role of dose escalated SABR beyond 37.5 Gy in high-risk patients is yet to be explored completely. This led to development of pHART8 protocol in 2011, in which the entire prostate was contoured as CTV2; the inferior 1.5 cm of seminal vesicles (entire SVs if T3b) were contoured as the CTV1, with a 5-mm margin for PTV1 and PTV2. An optimized radiotherapy plan was developed to treat the PTV2 to 40 Gy in five fractions (EQD2 of 103 Gy with an α/β of 1.8) (7); PTV1 received 30 Gy in five fractions (equivalent to 62 Gy in 2 Gy fractions). ADT was stopped after 36 months (45).

Thirty patients have been treated and no acute grade 3 toxicities were observed.

### pHART11: High risk

The role of ENI in high-risk prostate cancer remains controversial (46–48). In most of the EORTC and RTOG trials, the pelvis received a dose of 45–50 Gy to cover occult metastatic disease in the lymph nodes.

As most of the studies were done in pre-IGRT era (and therefore substantial volumes of pelvic nodal tissue may have been missed using standard bony landmarks), there is a requirement to evaluate the role of pelvic RT in the era of technical advances (49). RTOG 0924 study evaluating the role of ENI is ongoing.

Katz et al. have treated around 100 high-risk pts with either SABR alone (35–36.25 Gy in five fractions) or 63–66 Gy in 28 fractions (18–21 Gy in three fractions – SABR prostate boost), simultaneously delivering 45 Gy in 25 fractions to the pelvic lymph node targets. At a median follow-up of 5 years, there was no difference in bRFS or GU toxicity (44). Lack of benefit with the addition of EBRT could be due to the small sample size in each cohort (SABR-52pts, EBRT + SABR-45pts). Grade 2 GI toxicity was higher in patients who received pelvic EBRT (13.3% vs. 0, *p* = 0.0019). A 3D conformal technique was used to treat pelvic lymph nodes and this might explain the increase in bowel toxicity for this cohort. The reduction in toxicity with the change in radiation technique from 3D conformal to IMRT was demonstrated nicely in out phart 2 protocol (12). The experience with using 40 Gy to the prostate while treating the pelvic lymph nodes in five fractions is limited.

This led to the inception of pHART11 (SATURN) protocol. In this study, VMAT was used to treat CTV1 to 25 Gy and CTV2 to 40 Gy in five (weekly) fractions concomitantly, using daily CBCT pre- and post-treatment. PTV1 (23.75 Gy in five fractions) included a 6-mm margin around the CTV nodes (50) and seminal vesicles while the CTV2 (prostate) with a 3-mm margin was contoured as PTV2 (33.25 Gy in five actions-PTV dose in phart 3 study) (34). This protocol completed accrual in September 2014 and the acute toxicity results will be presented early 2015.

## Discussion

Given the success of SABR in treating lung cancer (51, 52), it is now being evaluated to treat other sites including spine, liver, and prostate.

US-based SABR consortium reported outcomes in 1100 localized prostate cancer patients treated with robotic non-coplanar SABR to a median dose of 36.25 Gy (range 35–40 Gy) in four to five fractions. At a median follow up of 36 months, the 5-year bRFS was 95, 84, and 81% for low, intermediate, and high-risk patients, respectively (53). As many patients experience benign bounce post-treatment, bRFS appears to be better for patients with longer follow-up: 99% for low risk, 93% for intermediate risk (22).

Prostate SABR trials have shown late grade 3 GI and urinary toxicities to be 1–3% (30). A QOL analysis for the 864 patients in the multi-institutional cohort showed a transient decline in the urinary and bowel domains within the first 3 months after SABR, which returned to baseline within 6 months. Sexual QOL decline was noticed within the first 9 months, irrespective of the use of ADT or age (53).

Given that the majority of local relapses occur at the site of primary tumor following radiotherapy for prostate cancer (54, 55), and to minimize the QOL decrements associated with whole gland boost, the next step would be dose painting with focal boost to the dominant nodule. A planning study by Udrescu et al., demonstrated a significant improvement in the bladder and rectal dosimetry in the MRI based focal boost plan when compared to the whole gland boost (56). In the study by Aluwini et al., 50 low- and intermediate-risk prostate cancer patients were treated with SABR to a total dose of 38 Gy delivered in four daily fractions and an integrated boost of up to 11 Gy per fraction was applied to the dominant lesion visible on MRI. At a median follow up of 23 months; acute Grade 3 GI and GU toxicity was 2 and 8%, and late Grade 3 GI and GU toxicity was 0 and 6%, respectively (57). This remains experimental and is being tested in multiple phase II studies. In these studies prostate and the visible dominant lesion will receive a dose anywhere between 36.25–45 Gy and 38–50 Gy, respectively (clinical trials.gov- NCT01409473, NCT01856855, NCT02145494, NCT01976962).

Other possibilities of focal dose escalation in the investigational setting include the utility of (11) C-choline PET for dose painting the dominant nodule (58).

## Conclusion

Stereotactic ablative body radiotherapy appears to be promising in low- and intermediate-risk prostate cancer and remains experimental in high-risk cohort with uncertainties about radiotherapy dose, ENI, and the duration of ADT. Studies showing that SABR can be safely delivered using a gantry-based platform (22) make it feasible to utilize this technique in public health care models as it is also economically valuable (59).

## Conflict of Interest Statement

Dr. Loblaw has received travel support and an honorarium from Elekta. The other co-authors declare that the research was conducted in the absence of any commercial or financial relationships that could be construed as a potential conflict of interest.
